# PPA1 Promotes Breast Cancer Proliferation and Metastasis Through PI3K/AKT/GSK3β Signaling Pathway

**DOI:** 10.3389/fcell.2021.730558

**Published:** 2021-09-14

**Authors:** Chunlei Guo, Shuang Li, Ang Liang, Mengchao Cui, Yunwei Lou, Hui Wang

**Affiliations:** ^1^Henan Key Laboratory of Immunology and Targeted Drugs, School of Laboratory Medicine, Xinxiang Medical University, Xinxiang, China; ^2^Henan Collaborative Innovation Center of Molecular Diagnosis and Laboratory Medicine, Xinxiang Medical University, Xinxiang, China; ^3^School of Nursing, Xinxiang Medical University, Xinxiang, China

**Keywords:** PPA1, breast cancer, EMT, metastasis, proliferation

## Abstract

Breast cancer is the most common malignancy among women. Inorganic pyrophosphatase 1 (PPA1) is a multifunctional protein involved in the development of several tumors. However, the role of PPA1 in breast cancer progression remains unclear. In this study, we found that PPA1 was highly expressed in breast cancer compared to its levels in normal breast tissue and that it was correlated with breast cancer clinicopathological characteristics, as well as poor survival in breast cancer patients. Silencing PPA1 restrained breast cancer proliferation and metastasis by regulating Slug-mediated epithelial-mesenchymal transition (EMT). Opposite results were observed following PPA1 overexpression. In addition, investigation of the underlying mechanism demonstrated that PPA1 ablation led to decrease phosphatidylinositol 3 kinase (PI3K) phosphorylation levels and attenuate phosphorylated AKT and glycogen synthase kinase-3 β (GSK3β), while ectopic PPA1 expression had the opposite effects. Moreover, PI3K inhibitors suppress the signaling pathways mediating the effects of PPA1 on breast cancer, resulting in tumor growth and metastasis suppression *in vitro* and *in vivo*. In summary, our results verify that PPA1 can act as an activator of PI3K/AKT/GSK3β/Slug-mediated breast cancer progression and that it is a potential therapeutic target for the inhibition of tumor progression.

## Introduction

Breast cancer is the most common female malignancy and a major cause of cancer-related mortality among women worldwide (Li et al., 2021; Siegel et al., 2021). Globally, although remarkable advancements have been achieved in the detection and treatment of breast cancer, the lethality is still severe (Cai et al., 2018; Fan et al., 2020).

The high mortality of breast cancer patients is mainly attributed to distal metastases, such as lung, bone, and lymph nodes metastases (Davis et al., 2020). Metastasis is a multistep process that includes tumor cells epithelial-mesenchymal transition (EMT), cytoskeleton reorganization, and microenvironment remodeling (Sleeman and Thiery, 2011; Cui et al., 2021). It is imperative to understand the detailed molecular mechanism involved in EMT for efficient identification of novel therapeutic targets and the development of effective treatment strategies for breast cancer.

The EMT is a complicated development process in which epithelial cells get deprived of polarity and transform into highly motile mesenchymal cells (Pastushenko et al., 2018; Xu et al., 2020). Profound changes in cell motility and morphology enable cells to travel long distances and integrate into surrounding tissues or remote organs (Chaffer et al., 2016). Furthermore, EMT contributes to tumor progression by granting aggressive traits to tumor cells, such as cell migration and other invasive properties that facilitate them departure from the epithelial cell community and arrival at the site of future migration (Popper, 2020). Therefore, EMT plays an important role in tumor metastasis.

Inorganic pyrophosphatase 1 (PPA1) catalyzes the hydrolysis of inorganic pyrophosphate (PPi) into inorganic phosphates (Pi). Therefore, it plays critical roles in multiple cellular metabolic processes, such as nucleic acid, proteins and carbohydrates synthesis, and neurite growth, owing to its highly catalytic property (Baykov et al., 1999). Meanwhile, it also provides extra energy source besides ATP (Li et al., 2018), which is indispensable for accelerating tumor growth and development (Yazdani et al., 2019).

Recently, proteomic studies have demonstrated that PPA1 was overexpressed in various types of malignancies, such as colorectal cancer (Tomonaga et al., 2004), ovarian cancer (Giri et al., 2014), lung adenocarcinoma (Chen et al., 2002), hepatocellular carcinoma (Megger et al., 2013), and prostate cancer (Lexander et al., 2005), compared to its levels in normal tissues. Notably, PPA1 was considered a negative prognostic marker of gastric cancer and participated in cancer-related metabolic alterations (Yang et al., 2015). Moreover, Bodnar et al. (2016) reported that PPA1 may be considered as biomarker of metastasis in laryngeal squamous cell carcinoma, and Luo et al. (2019) illustrated that ectopic PPA1 expression ameliorated cell proliferation properties in non-small cell lung cancer. Therefore, the association of PPA1 to cell proliferation and metastasis in cancer is undisputable. However, the significance of PPA1 expression and its role in breast cancer remains unclear. The mechanisms underlying these potential functions also require further investigation.

Our study demonstrated that PPA1 plays an important role in breast cancer progression for the first time and we extensively explored its functions *in vitro* and *in vivo*. We found that PPA1 was significantly upregulated in the tissues of patients with breast cancer, and its expression was correlated with clinicopathological characteristics. Meanwhile, cell-based and mouse models studies indicated that PPA1 promoted proliferation, migration, and invasion of breast cancer. Moreover, we provided evidence for its effect on EMT process. In particular, we found that overexpression of PPA1 enhanced EMT under Slug stimulation. Finally, we investigated the mechanism of action of PPA1 in breast cancer progression. Taken together, these findings provide novel insight into how PPA1 regulates tumor metastasis, suggesting its potential as a therapeutic target in breast cancer.

## Materials and Methods

### Cell Culture

Human breast cancer cell line T47D and MDA-MB-231 were purchased from American Type Culture Collection (ATCC). T47D cells were cultured in high glucose Dulbecco’s Modified Eagle’s Medium (Gibco, Waltham, MA, United States) supplemented with 10% fetal bovine serum (FBS), 100 U/mL penicillin, 0.1 mg/mL streptomycin and 1% non-essential amino acid (NEAA) solution (Gibco). And the cells were maintained in the incubator at 37°C and in the presence of 5% CO_2_. MDA-MB-231 cells were cultured in L-15 medium (Gibco, Waltham, MA, United States) supplemented with 10% FBS, 100 U/mL penicillin, 0.1 mg/mL streptomycin. The cells were maintained in the incubator at 37°C.

### Vector Construction and Stable Cell Line Establishment

In order to better analyze the correlation between PPA1 and breast cancer progression, we hope to choose the cell line with relatively lower expression level at basal status for overexpression assays and with relatively higher expression level for knockdown assays. Meanwhile, we would like to select the cell line with high malignancy and aggressively metastatic characteristics for overexpression, and study the role of PPA1 in different molecular subtypes of breast cancer. For above considerations, we choose T47D for knockdown and MDA-MB-231 for overexpression assays.

To stably knockdown PPA1 in breast cancer cells, T47D cells were infected with lentivirus carrying pLV-H1-shPPA1-puro or pLV-H1-shRNA-scramble-puro plasmid, then treated with puromycin to obtain the stable cell line with PPA1 silencing (shPPA1) and the shRNA control. The sequences of shRNAs were: shPPA1#1: GCTACTGTGGACTGGTTTA; shPPA1#2: GGAATCAGTTGCATGAATA; shRNA control: GCTACACTATCGAGCAATT.

For the stable overexpression of PPA1 in mammalian cells, the cDNA of *PPA1* was inserted into the pLV-EF1α-MCS-IRES-Bsd plasmid. MDA-MB-231 cells were infected with lentivirus carrying the recombinant plasmid and empty plasmid which was used as the control. Cells were selected using blasticidin to generate stable cell lines with PPA1 overexpression and their control.

### Western Blotting

Cell lysates were prepared from T47D and MDA-MB-231 with RIPA buffer containing protease inhibitor cocktail, phosphatase inhibitor cocktails 2 and 3 (Sigma-Aldrich, St Louis, MO, United States). Proteins (20–40 μg) were loaded onto 10–15% Tris-Acrylamide gels and blotted with primary antibodies that included: anti-PPA1 (Cat. # HPA019878, Sigma, St. Louis MO), anti-β-actin (sc-47778, Santa Cruz Biotechnology Inc., Santa Cruz, CA), anti-E-cadherin, N-cadherin, Vimentin, ZO-1, Claudin-1, Slug, PI3K, p-PI3K (Tyr458), AKT, p-AKT (Ser473), p-GSK3β (Ser9), P38, p-P38 (Thr180/Tyr182), JNK, p-JNK (Thr183/Tyr185), (Cat. # 3195, Cat. # 13116, Cat. # 5741, Cat. # 8193, Cat. # 13255, Cat. # 9585, Cat. # 4292, Cat. # 4228, Cat. # 4691, Cat. # 4060, Cat. # 5558, Cat. # 8690, Cat. # 9216, Cat. # 9252, Cat. # 4668, Cell Signal Technology Inc., Danvers, MA, United States), anti-Ki67 (Cat. # ab16667, Abcam Inc., Cambridge, United Kingdom), and followed by incubation with horseradish peroxidase-conjugated secondary antibodies (Santa Cruz Biotechnology, Inc., Santa Cruz, CA). Images were acquired using the Amersham Imager 600RGB detection system (GE Healthcare).

### Real-Time PCR

Total RNA was extracted from cells using the TRIZOL reagent and reverse transcription was performed using the M-MLV reverse transcriptase (Promega, Madison, MI). Real-time PCR was performed using the TransStart Green qPCR Super Mix Kit (TransGen Biotech, Beijing, China). The 2^–ΔΔ^ ^Ct^ method was used to obtain the relative fold changes. The primers for human *GAPDH* were CTCTGATTTGGTCGTATTGGG and TGGAAGATGGTGATGGGATT. The primers for human *PPA1* were CGCTATGTTGCGAATTTGTTC and CCAGTATGTTTATCATTGTGCC.

### Transwell Assay

Transwell chambers were pre-coated with Matrigel. The bottom chamber was filled with culture medium containing 10% FBS. Equal amounts of cells were plated in the upper chamber in serum-free medium. After 12–24 h, the invasive cells were fixed and stained with 0.5% crystal violet. Then, we used microscope to observe the invasive cells.

### Wound Healing Assay

For each test, T47D or MDA-MB-231 cells were seeded in plates. When the cells reached full confluence, a “wound” was created in the middle of the culture plate and the concentration of the serum in culture medium was changed from 10 to 1% to avoid the influence of cell growth rate on wound healing. The wound healing process was recorded at 0, 12, and 24 h after wound creation. The wound healing rate was quantified as the distance between the wound recovered compared to that of the original wound.

### Cell Proliferation Assay

Cell proliferation was measured using the Cell Counting Kit-8 (CCK-8). Equal amounts of cells were seeded in 96-well plates. At designated time points (0, 24, 48, 72, and 96 h), the CCK-8 reagent was added to each well and then incubated for 2 h. Finally, the absorbance was measured at 450 nm.

### Immunohistochemistry and Tissue Microarrays

The tissue microarray used for the analysis of PPA1 expression in breast cancer were purchased from Biomax Inc. (Cat. # BR2086, Rockville, United States). Immunohistochemistry (IHC) was performed on tissue microarray using antibodies against PPA1 at 1:200 dilution. The expression level of PPA1 was evaluated according to the percentage of positive cells and its staining intensity in each tumor tissue. The percentage of positive cells was separated into < 10%, 10%–29%, 30%–49%, and ≥ 50% subgroups, which were scored as 1, 2, 3, and 4, respectively. The staining intensities were evaluated as negative, weak, moderate, and strong, which were scored as 1, 2, 3, and 4, respectively. The cell percentage score and staining intensity score were multiplied to obtain the IHC score.

### Immunofluorescence

Cells grown on glass slides were fixed in 4% paraformaldehyde and labeled with primary antibodies overnight at 4°C, followed by incubation with Alexa Fluor 488 goat anti-mouse IgG (Invitrogen) or Alexa Fluor 594 goat anti-rabbit IgG (Invitrogen) at RT for 1 h. The cells were then subjected to confocal imaging (Leica, Germany).

### Animal Study

All *in vivo* mouse experiments were approved by the Ethics Committee of Xinxiang Medical University. Female nude mice at 5–6 weeks old were separated randomly into four groups (*n* = 5 for each group). Cells were subcutaneously inoculated into the fourth mammary fat pad of each mouse. Tumor volume was measured using calipers and calculated using the formula: length × width^2^/2. Fifty days after inoculation, tumor and lung tissues were collected and subjected to IHC or hematoxylin and eosin (H&E) staining. For the inhibitor treatment assay, 10 days after tumor cells injection, mice were treated with PI3K inhibitor (LY294002, 70 mg/kg) every 2 days, while dimethyl sulfoxide (DMSO) was used as the control.

### Patient Datasets

Survival analyses were conducted using an online tool.^1^ Patients with breast cancer (*n* = 1,070) were selected for the overall survival assay. The log-rank test was computed automatically.

### Statistical Analysis

Values were expressed as the mean ± SEM. Statistical significance was determined using the Student’s *t*-test. A value of *p* < 0.05 was considered statistically significant. ^∗^ indicates significant difference with *p* < 0.05, ^∗∗^ indicates significant difference with *p* < 0.01, ^∗∗∗^ indicates significant difference with *p* < 0.001.

## Results

### PPA1 Is Highly Expressed in Human Breast Cancer and Correlates With TNM Stage and Histological Grade

**FIGURE 1:**
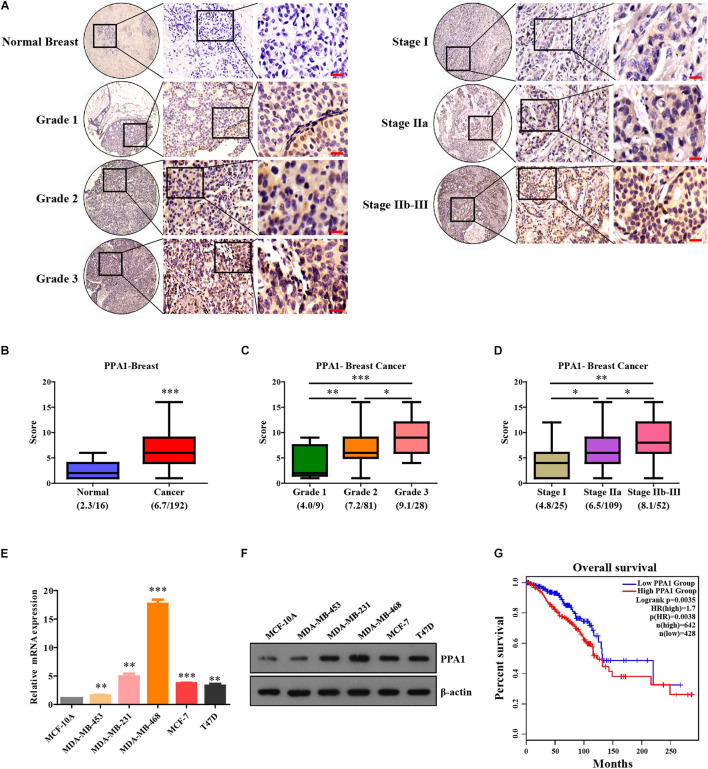
PPA1 is highly expressed in breast cancer and correlates with TNM stage and histological grade. **(A)** Immunohistochemical analysis of PPA1 protein expression using human breast cancer tissue microarray. Scale bars: 20 μm. **(B)** Statistical analyses of the IHC scores of PPA1 in the breast cancer tissue microarray. **(C)** Quantification of PPA1 immunostaining and pathological grades. **(D)** Quantification of PPA1 immunostaining and clinical stages. **(E)** Real-time PCR showing the expression level of PPA1 in the normal breast cell line compared to that in breast cancer cell lines. **(F)** PPA1 protein expression examined using western blotting in the normal breast cell line compared to that in breast cancer cell lines. **(G)** Correlation of PPA1 expression in breast cancer patients with the overall survival rate. ^∗^*p* < 0.05, ^∗∗^*p* < 0.01, and ^∗∗∗^*p* < 0.001.

In order to verify the clinical significance of PPA1 in breast cancer, we first examined PPA1 expression in tissue microarray containing human normal/para-carcinoma breast tissues and breast tumors with different TNM stage and histological grade by IHC. The results indicated that PPA1 was highly expressed in tumor tissues compared to normal/para-carcinoma tissues (Figures 1A,B). In addition, since the pathological grade and clinical stage of tumor were closely correlate with tumor malignancy and progression, we investigated the associations between PPA1 expression and clinicopathological characteristics. We found that PPA1 expression was positively correlated with TNM stage and histological grade of human breast cancer (Figures 1C,D). Moreover, the increased expression of PPA1 was also observed in breast cancer cell lines compared to that in the normal breast cell line (Figures 1E,F). Furthermore, we analyzed the correlation between the expression of PPA1 and patient overall survival (OS), and found that high PPA1 expression was associated with poor clinical outcomes in patients with breast cancer (Figure 1G). Taken together, these results indicate that PPA1 may play a vital role in breast cancer development, and that it may be a diagnostic marker for tumor progression.

### PPA1 Promotes Breast Cancer Cell Proliferation, Migration, and Invasion

To elucidate the function of PPA1 in breast cancer, we first silencing PPA1 in T47D cells by stable expression of control or two PPA1-targeting shRNAs. Meanwhile, we established stable MDA-MB-231 cell lines that overexpressed PPA1.

**FIGURE 2:**
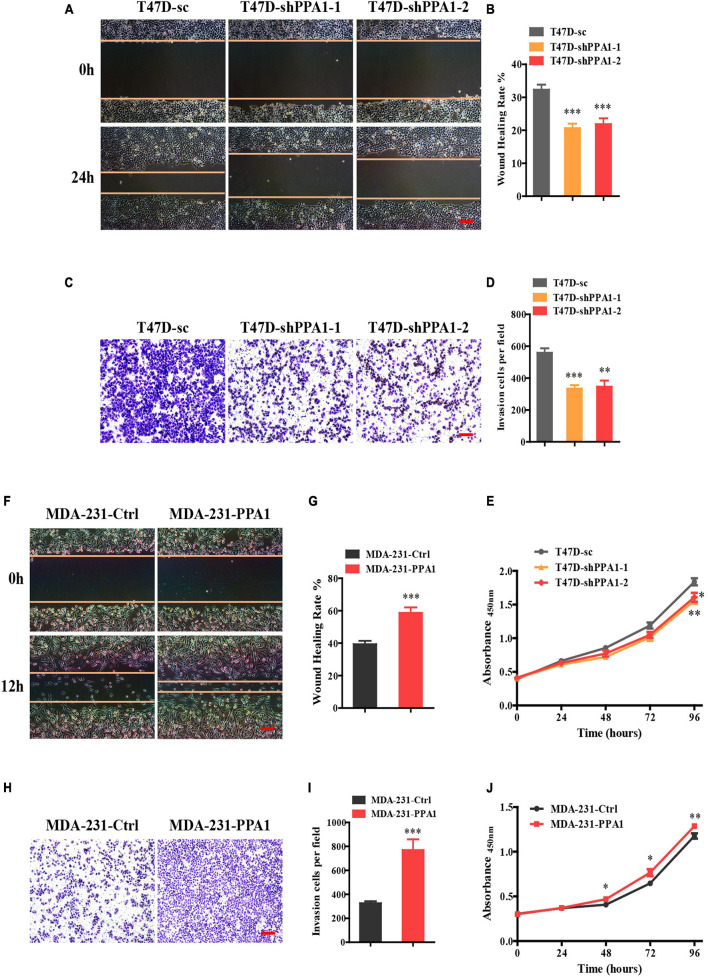
PPA1 promotes proliferation, migration, and invasion of breast cancer cells. **(A)** Silencing PPA1 suppresses breast cancer cell migration as demonstrated in the wound healing assay. Scale bars: 100 μm. **(B)** Quantitative analysis of the wound healing rate. **(C)** Transwell assay showing that silencing PPA1 decreases cell invasion. Scale bars: 100 μm. **(D)** Quantitative analysis of invasion cells. **(E)** PPA1 knockdown inhibits breast cancer cell proliferation in T47D cells. **(F–I)** Ectopic PPA1 facilitates tumor migration and invasion in MDA-MB-231 cells. Scale bars: 100 μm. **(J)** PPA1 overexpression increases cell proliferation. ^∗^*p* < 0.05, ^∗∗^*p* < 0.01, and ^∗∗∗^*p* < 0.001.

Wound healing assays were performed to detect the role of PPA1 in migration. As shown in Figure 2A, although gap-filling was significantly retarded in T47D-shPPA1 cells, the control cells migrated and almost filled the gap at 24 h after wounding (Figures 2A,B), indicating that silencing PPA1 suppressed the migration properties of T47D cells. Moreover, the transwell assay also demonstrated that PPA1 knockdown reduced the cell invasion properties of T47D-shPPA1 cells compared to that in the control group (Figures 2C,D). The CCK-8 results revealed that silencing PPA1 inhibited cell proliferation (Figure 2E).

To strengthen our conclusions, we examined the effect of PPA1 overexpression in MDA-MB-231 cells. Consistently, wound healing and transwell assay confirmed that ectopic PPA1 facilitated breast cancer cell migration and invasion (Figures 2F–I). Furthermore, PPA1 overexpression increased cell proliferation (Figure 2J). Taken together, these findings verified that PPA1 promoted proliferation, migration, and invasion of breast cancer cells.

### PPA1 Triggers Breast Cancer EMT

**FIGURE 3:**
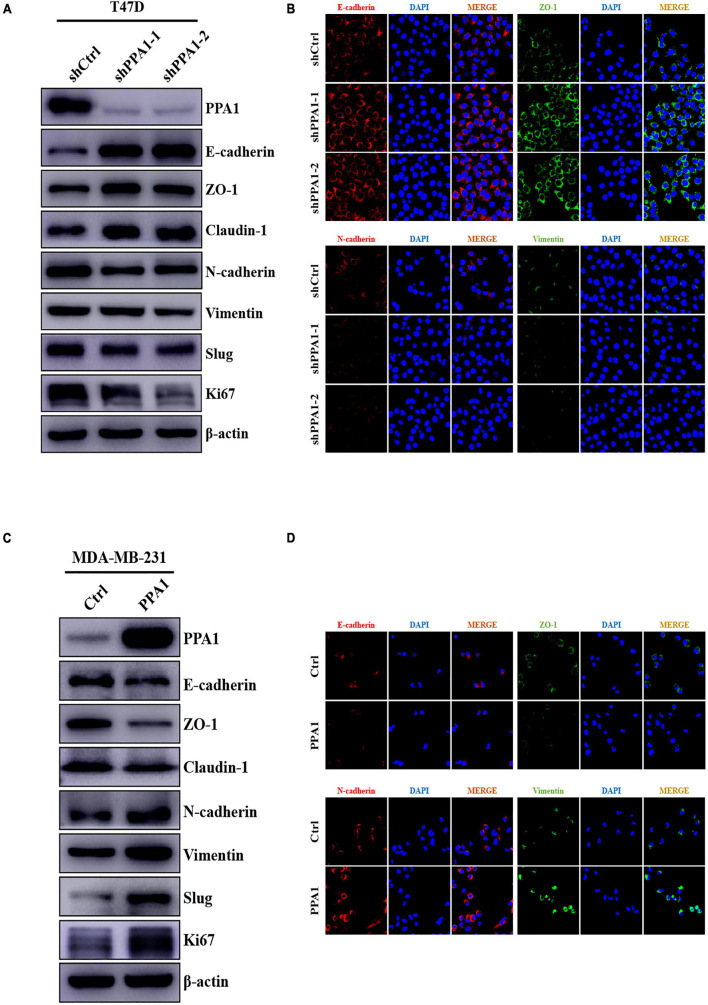
PPA1 triggers EMT in breast cancer. **(A,C)** Western blot analysis showing the protein expression of EMT-related markers after PPA1 knockdown in T47D cells and overexpression PPA1 in MDA-MB-231 cells, respectively; **(B,D)** Representative immunofluorescence staining images of EMT-related markers in T47D and MDA-MB-231 cells.

To tested the regulatory effect of PPA1 on EMT, we first examined the expression of EMT-related proteins by western blot. We observed that PPA1 deficiency contributed to an epithelial phenotype as the expression levels of E-cadherin, ZO-1, and Claudin-1 were elevated, and those of the mesenchymal marker proteins Vimentin and N-cadherin were diminished in PPA1-deficient cells compared to those of the control group (Figure 3A), while ectopic PPA1 contribute to mesenchymal characteristic by attenuating expression levels of E-cadherin, ZO-1 and Claudin-1, and augmenting levels of Vimentin and N-cadherin (Figure 3C), indicating the EMT-promoting effects of PPA1. Consistently, this was further supported by the immunofluorescence results, which revealed an elevation in the intensity of the E-cadherin and ZO-1 signals in T47D-shPPA1 cells, and a decrease in the intensity of these signals in MDA-MB-231-PPA1 cells. Meanwhile, the expression of N-cadherin and Vimentin was attenuated in T47D-shPPA1 cells, and enhanced in MDA-MB-231-PPA1 cells (Figures 3B,D).

In addition, several transcriptional factors, including Slug, Twist, and ZEB1, function as molecular switches in the EMT program. Especially Slug, a major EMT inducer, is closely associated with tumor metastasis. To investigate which transcriptional factors mediate the regulatory effects of PPA1 on EMT, we used western blotting and found that the protein expression levels of Slug decreased in two T47D-shPPA1 cells, but increased in MDA-MB-231-PPA1 cells (Figures 3A,C). Meanwhile, PPA1 knockdown or overexpression elicited no change in ZEB1, Twist, and Snail expression. These results verified that PPA1 played a vital role in triggering EMT and revealed the crucial function of Slug in mediating EMT promotion effect of PPA1.

### PPA1 Facilitates Breast Cancer Progression and EMT Through the PI3K/AKT/GSK3β Pathway

**FIGURE 4:**
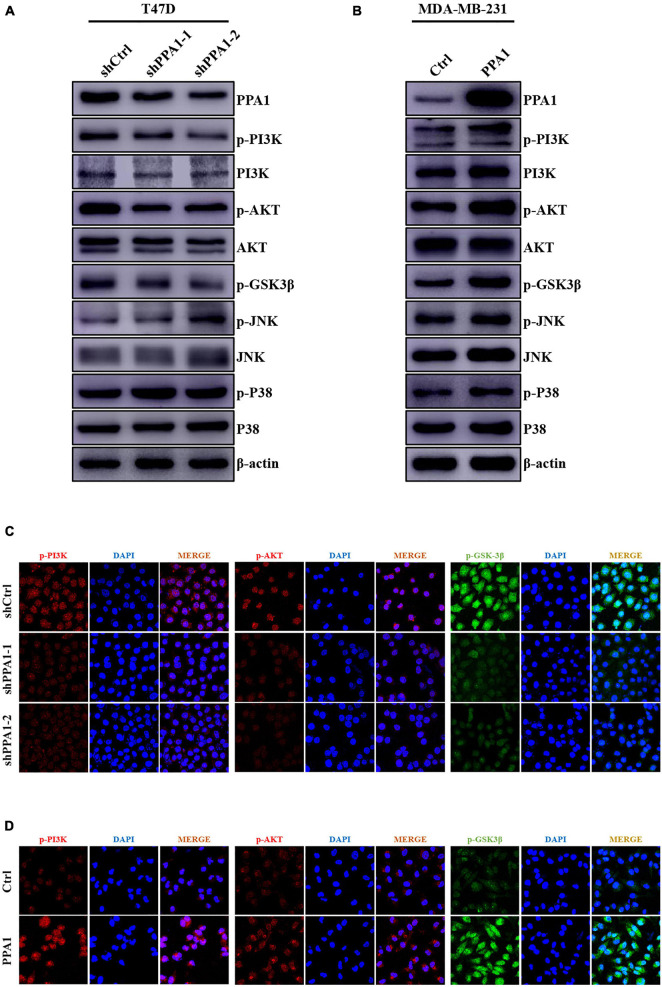
PPA1 facilitates breast cancer progression and EMT through the PI3K/AKT/GSK3β signaling. **(A,B)** Western blot analysis showing the protein expression of JNK, p-JNK, P38, p-P38, and PI3K/AKT/GSK3β signaling molecules after PPA1 knockdown in T47D cells and overexpression PPA1 in MDA-MB-231 cells, respectively; **(C,D)** Representative immunofluorescence staining images of p-PI3K, p-AKT, and p-GSK3β in T47D and MDA-MB-231 cells.

To further investigate the mechanism underlying the regulatory effects of PPA1 on breast cancer progression and EMT, we first performed western blot and the results demonstrated that silencing PPA1 restrained the phosphorylation levels of PI3K, AKT, and GSK3β. In contrast, ectopic PPA1 augmented the expression of p-PI3K (Tyr458), p-AKT (Ser473), and p-GSK3β (Ser9) (Figures 4A,B). PPA1 also could induce more phosphorylated GSK3β accumulation in the cytoplasm and less in the nucleus (Supplementary Figures 1A,B). However, significantly changes in JNK, p-JNK (Thr183/Tyr185), P38, and p-P38 (Thr180/Tyr182) were not as evident (Figures 4A,B). Consistently, this was further supported by immunofluorescence assay results (Figures 4C,D). Collectively, these findings indicated that PPA1 facilitated breast cancer progression and EMT via activating PI3K/AKT/GSK3β signaling pathway.

### Inhibitors Targeted PPA1 Mediated Signaling Pathway Suppresses Breast Cancer Progression and EMT *in vitro*

**FIGURE 5:**
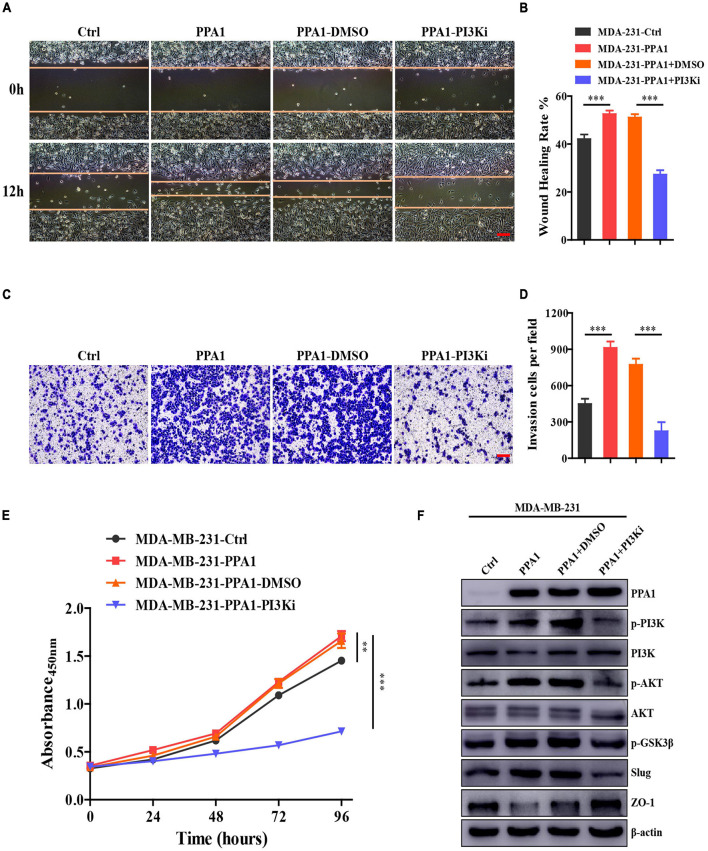
Inhibitors of the PI3K/AKT/GSK3β signaling pathway can restore PPA1-induced breast cancer cell proliferation, invasion and migration. **(A,B)** Cell migration are detected by wound healing assay after cells are treated with PI3K inhibitor. Scale bars: 100 μm. **(C,D)** Cell invasion are detected by transwell assay after cells are treated. Scale bars: 100 μm. **(E)** Cell proliferation examined by CCK-8 assay. **(F)** Western blot analysis showing the protein expression of PPA1, p-PI3K, p-AKT, p-GSK3β and EMT markers in PPA1-expressing cells treated with the PI3K inhibitor. *p < 0.05, **p < 0.01, and ***p < 0.001.

To gain insights into the importance of PPA1 mediated signaling pathway in promoting the breast cancer progression and EMT, we investigated the effects of PI3K inhibitor (LY294002) in MDA-MB-231-PPA1 cells. We verified that the PI3K inhibitor reversed the proliferation, migration and invasion promoting effects of PPA1 in MDA-MB-231 cells (Figures 5A–E). Consistently, the treatment of the cells with PI3K inhibitor rescued expression levels of ZO-1, attenuated Slug activity, and blunted PPA1-induced PI3K/AKT/GSK3β signaling pathway activation (Figure 5F). Taken together, these results confirmed that inhibitors targeted PPA1 mediated signaling pathway suppressed breast cancer progression and EMT *in vitro*.

### PPA1 Promotes Tumor Growth and Metastasis via the PI3K/AKT/GSK3β Signaling Pathway *in vivo*

**FIGURE 6:**
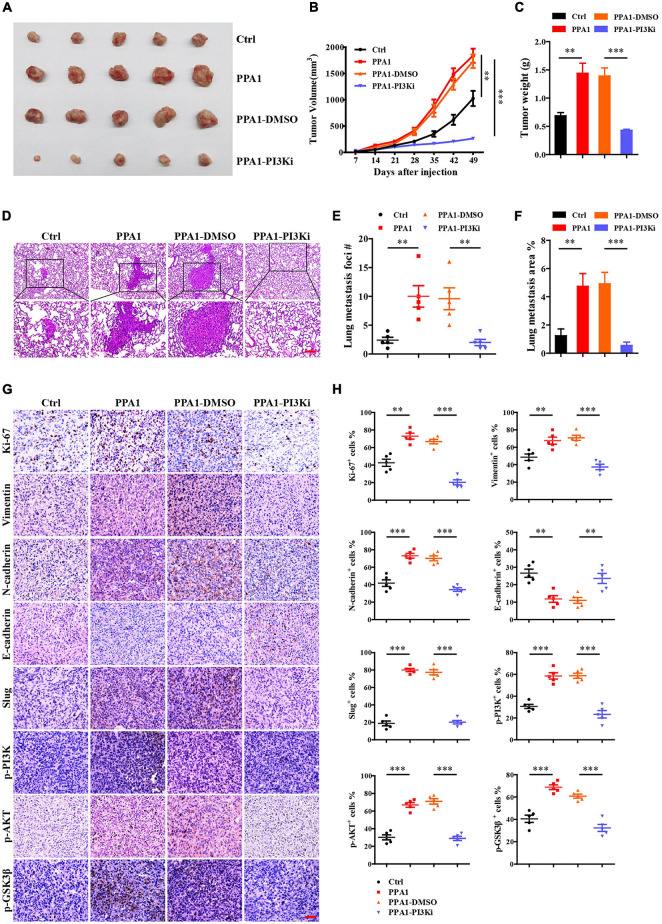
PPA1 promotes tumor growth and metastasis via the PI3K/AKT/GSK3β signaling *in vivo*. **(A–C)** Ectopic PPA1 promotes tumor growth, which is reversed by the PI3K inhibitor. **(D)** H&E staining is used to analyze lung metastasis from the indicated mice. Scale bars: 100 μm. **(E,F)** Quantification of metastases (*n* = 5). **(G,H)** Immunohistochemistry staining of p-PI3K, p-AKT, p-GSK3β, and EMT markers in tumor tissue sections and quantification (*n* = 5). Scale bars: 50 μm. ^∗^*p* < 0.05, ^∗∗^*p* < 0.01, and ^∗∗∗^*p* < 0.001.

To evaluate the function of PPA1 in breast cancer progression *in vivo*, we performed xenograft experiments using MDA-MB-231 cells. To this end, stable MDA-MB-231-control and MDA-MB-231-PPA1 cells were injected into the fourth mammary fat pad of mice. We verified that ectopic PPA1 significantly promoted tumor growth, which could be reversed by the PI3K inhibitor (Figures 6A–C). In addition, we observed that more PPA1-upregulated cells metastasized to the lung and initiated secondary tumor, which was also rescued by the PI3K inhibitor (Figures 6D–F). Furthermore, immunohistochemical staining demonstrated that PPA1 upregulated boosted the expression of mesenchymal effect (N-cadherin, Vimentin) and reduced epithelial protein expression (E-cadherin). Meanwhile, the levels of Ki-67, Slug, p-PI3K (Tyr458), p-AKT (Ser473) and p-GSK3β (Ser9) were elevated in tumor tissues (Figures 6G,H). However, the PI3K inhibitor reversed the expression levels of EMT markers and modulated the activation of PI3K/AKT/GSK3β pathway (Figures 6G,H), demonstrating that inhibitors targeted PPA1 mediated signaling pathways suppressed breast cancer progression. Taken together, these results confirmed that PPA1 promoted tumor growth and metastasis via the PI3K/AKT/GSK3β signaling *in vivo*.

In summary, our study demonstrated a critical role of PPA1 in mediating PI3K/AKT/GSK3β signaling-induced tumor progression, which could be a useful target to prevent breast cancer.

### Proposed Model of PPA1 in Breast Cancer Progression

**FIGURE 7:**
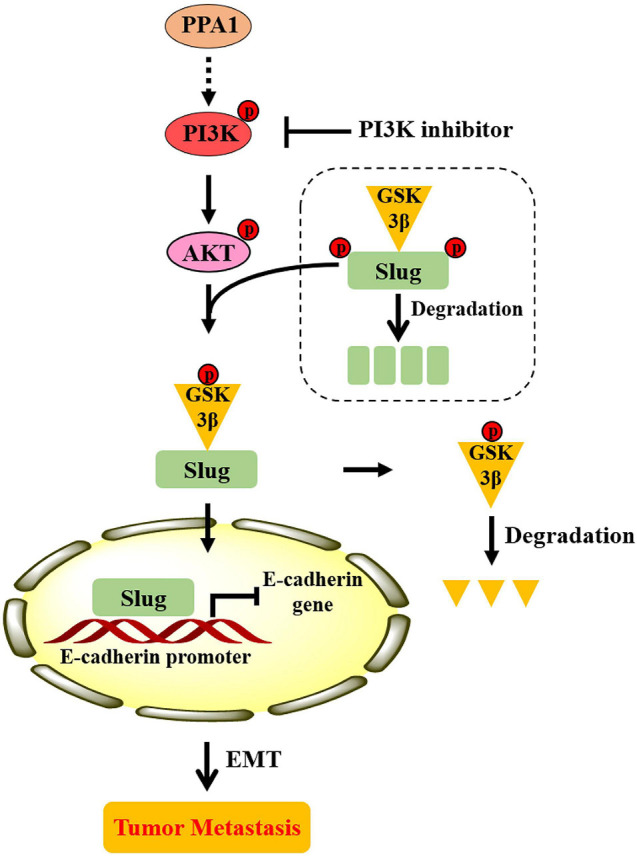
Proposed model illustrating the role of PPA1 in promoting breast cancer proliferation and metastasis.

Based on the totality of our findings, we propose the following model: PPA1 activates PI3K/AKT signaling, which enhances GSK3β phosphorylation, thereby maintains Slug stability and promotes Slug nuclear translocation. Slug then leads to the repression of E-cadherin expression, which promotes the EMT process and tumor metastasis (Figure 7).

## Discussion

Breast cancer is a heterogeneous, malignant, and life-threatening tumor that affects women worldwide. The prognosis of breast cancer patients has not markedly improved because of the high frequency of metastasis, relapse, and drug resistance. Hence, the identification of cancer progression targets may contribute to the development of more effective diagnostic and therapeutic strategies.

In this study, we determined the function of PPA1 in breast cancer and identified the potential molecular mechanism. Our results demonstrated that PPA1 was overexpressed in breast cancer and its expression was significantly correlates with clinical progression. Furthermore, we verified that PPA1 overexpression promoted proliferation, migration, invasion, and EMT *in vitro* and *in vivo*. The opposite effects were observed when PPA1 was silenced. Most importantly, we revealed the underlying mechanism that PPA1 regulated breast cancer progression and EMT through the PI3K/AKT/GSK3β pathway.

Metastasis is the major cause of high mortality rate in patients with breast cancer. There are multiple steps in the metastatic cascade, including cancer cells are divorced from primary tumor, invasion into stromal tissues, intravasation into lymphatic or blood vessels, survival within the circulation, extravasation, metastatic colonization and growth at secondary sites (Pantel and Brakenhoff, 2004). As a hallmark of metastasis, EMT is involved in the metastatic cascade of most carcinomas.

EMT is a highly conserved process that entails molecular reprogramming and is characterized by the phenotypic transformation of immobile epithelial cells to migratory mesenchymal cells. Previous studies have verified that activation of the EMT in primary breast cancer is indispensable for tumor cell dissemination to the distant sites, such as lung, bone, and lymph nodes (Ocana et al., 2012; Liu et al., 2019). Consequently, in order to develop effective therapeutic strategies for suppressing tumor metastasis and improve treatment outcomes, it is indispensable for us to investigate the mechanism of EMT. In our study, we identified that PPA1 promoted breast cancer metastasis via Slug-mediated EMT, proceeding through the PI3K/AKT/GSK3β signaling pathway.

Several transcriptional factors, such as Slug, Twist, and ZEB1, have been reported to play a vital function in EMT (Kurppa et al., 2020; Dufourt et al., 2021; Huang et al., 2021). Slug is one of the critical regulators which lead to EMT and is closely related to cancer metastasis. It induces EMT by repressing the expression of E-cadherin (Rao et al., 2021). Slug is also involved in various cellular processes, including neural crest cell migration and mesoderm formation (Reece-Hoyes et al., 2009; Ke et al., 2019; Flach et al., 2021). Additionally, Slug plays an important role in tumor metastasis and recurrence. It has been reported that Slug is essential in the metastatic process of melanoma cells (Furst et al., 2019). Increased levels of Slug were associated with cancer recurrence (Schinke et al., 2021). In this study, we identified a novel mechanism by which PPA1 selectively up-regulates the expression of the EMT master regulator Slug to drive EMT and enhance tumor metastasis.

Many signaling pathways, including PI3K/AKT/GSK3β, Wnt/β-catenin, and JAK/STAT signaling, play a pivotal role in EMT promotion (Pan et al., 2020; Wang et al., 2020; Yu et al., 2021). The activation of the PI3K/AKT/GSK3β axis is emerging as a central feature of EMT. PI3K/AKT constitutive activation results in the repression of epithelial characteristics and induction of the expression of mesenchymal protein, that increased tumor motility, invasiveness, and metastatic potential. GSK3β is highly inactivated in cancers, and GSK3β-mediated phosphorylation of Slug facilitates Slug protein ubiquitylation and degradation. In contrast, the p-GSK3β (Ser9) level is associated with Slug expression and maintains Slug protein stability (Kao et al., 2014). The accumulation of non-degradable Slug may further lead to restrain the expression of E-cadherin and accelerate the EMT process. In this study, we demonstrated that PPA1 activated the PI3K/AKT pathway, which then enhanced GSK3β phosphorylation, thereby preventing Slug destruction. Taken together, we confirmed that PPA1 promoted breast cancer proliferation, migration, and invasion through PI3K/AKT/GSK3β signaling.

Furthermore, recent evidence has revealed that a variety of small molecule inhibitors that targeting the PI3K/AKT/GSK3β axis attenuated tumor progression (Zhang et al., 2020; Crabb et al., 2021). We observed that PI3K inhibitor (LY294002) reversed the metastasis-promotion effect of PPA1 and the EMT process by inhibiting PI3K/AKT/GSK3β signaling. In addition, Slug expression was also repressed, suggesting a role for this pathway in Slug induction.

However, how does PPA1 regulate PI3K and further activate PI3K/AKT/GSK3β phosphorylation cascades. In order to verify the relationship between PPA1 and PI3K, we performed immunoprecipitation assay and the result revealed that there was no interaction between PPA1 and PI3K. That indicates PI3K activation requires another protein. It was reported that FAM120A could recruit PI3K and functioned as a scaffold protein to enable phosphorylation and activation of PI3K by Src family kinases (Bartolomé et al., 2015). In addition, PI3K is composed of a catalytic subunit (p110) and a regulatory subunit (p85). As a key molecular target of PI3K, p85 plays an important role in the activation of the PI3K/AKT signaling pathway. The stem cell biomarker CD133 direct interaction with PI3K-p85, resulting in activation of PI3K/AKT pathway (Song et al., 2018). According to these results, we reckon that there will be one or more linking proteins between PPA1 and PI3K. Therefore, we will continue to identify the important interlinking protein and explore the molecular mechanism in the following research.

## Conclusion

In conclusion, we verify that PPA1 is highly expressed in breast cancer and that it is correlated with clinicopathological characteristics. PPA1 promotes proliferation, migration, and invasion of breast cancer through the PI3K/AKT/GSK3β pathway. Thus, PPA1 may serve as a potential marker for evaluating breast cancer progression, and targeting PPA1 may be an attractive therapeutic strategy in breast cancer to prevent tumor metastasis.

## Data Availability Statement

The original contributions presented in the study are included in the article/Supplementary Material, further inquiries can be directed to the corresponding author/s.

## Ethics Statement

The animal study was reviewed and approved by the Committee on the Ethics of Animal Experiments of Xinxiang Medical University.

## Author Contributions

HW designed the project and supervised the study. CG, SL, AL, and MC carried out the experiments. CG, SL, and AL analyzed the data. CG and AL drafted the manuscript. YL provided support with experimental techniques. All authors read and approved the final manuscript.

## Conflict of Interest

The authors declare that the research was conducted in the absence of any commercial or financial relationships that could be construed as a potential conflict of interest.

## Publisher’s Note

All claims expressed in this article are solely those of the authors and do not necessarily represent those of their affiliated organizations, or those of the publisher, the editors and the reviewers. Any product that may be evaluated in this article, or claim that may be made by its manufacturer, is not guaranteed or endorsed by the publisher.

## Footnotes

1
http://gepia2.cancer-pku.cn/#survival
