# Supplementary material for: PPA1 Promotes Breast Cancer Proliferation and Metastasis Through PI3K/AKT/GSK3β Signaling Pathway
Source: Front Cell Dev Biol. 2021 Sep 14;9:730558. doi: 10.3389/fcell.2021.730558 (PMC8476924; doi:10.3389/fcell.2021.730558)
Supplement: Supplementary file 1 [file Data_Sheet_1.pdf]

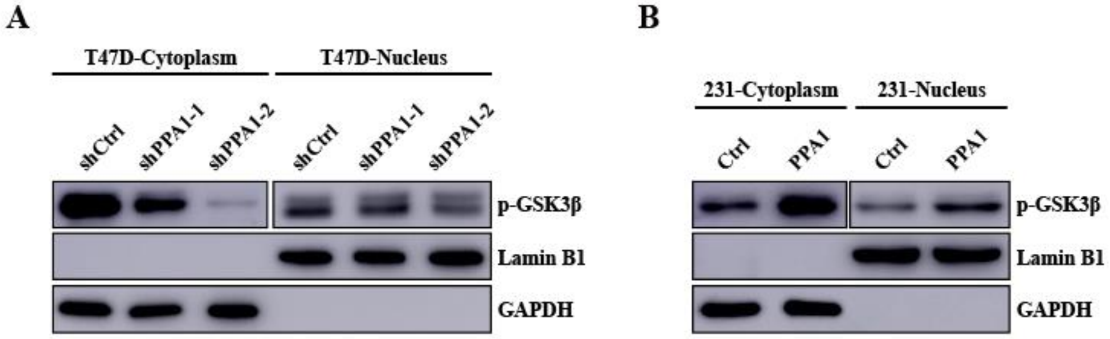

Figure S1. PPA1 regulates the expression of p-GSK3β in cytoplasm and nucleus. (A) Silencing PPA1 restrain the phosphorylation levels of GSK3β in T47D cells; (B) Ectopic PPA1 augment the expression of p-GSK3β in MDA-MB-231 cells.
